# Construction of a competence-based curriculum for internship in obstetrics and gynecology within the medical course at the Federal University of Ceará (Sobral campus): Construção do currículo por competências para o internato em obstetrícia e ginecologia do curso de medicina da Universidade Federal do Ceará (Campus de Sobral)

**DOI:** 10.1590/1516-3180.2014.0804872

**Published:** 2015-04-14

**Authors:** José Juvenal Linhares, Bárbara de Araújo Lima Dutra, Maycon Fellipe da Ponte, Luis Fernando Farah de Tofoli, Priscila Campos Távora, Filipe Sancho de Macedo, Guarany Mont’Alverne de Arruda

**Affiliations:** I MD, PhD. Assistant Professor, Discipline of Personal Development, Faculdade de Medicina da Universidade Federal do Ceará (FMUFC), Sobral, Ceará, Brazil.; II Medical Student, Universidade Federal do Ceará (UFC), Sobral, Ceará, Brazil.; III MD, PhD. Adjunct Professor, Discipline of Personal Development, Faculdade de Medicina da Universidade Federal do Ceará (FMUFC), Sobral, Ceará, Brazil.; IV MD. Assistant Professor, Discipline of Personal Development, Faculdade de Medicina da Universidade Federal do Ceará (FMUFC), Sobral, Ceará, Brazil.

**Keywords:** Internship and residency, Obstetrics, Gynecology, Clinical competence, General practitioners, Internato e residência, Obstetrícia, Ginecologia, Competência clínica, Clínicos gerais

## Abstract

**CONTEXT AND OBJECTIVE:**

This research project arose from a proposal made to the teachers by the students of a medical course at a federal university in Brazil, from their personal experiences regarding the skills and competencies that should be developed during the obstetrics and gynecology (OBG) stage of the internship. The objective here was to develop the matrix of skills necessary for training good general physicians in the medical course.

**DESIGN AND SETTING:**

Exploratory qualitative study conducted in a federal university in Brazil.

**METHODS:**

The basis for developing these competencies among OBG interns was “The Competency Matrix for Medical Internship” developed by Bollela and Machado. The instrument was presented to, analyzed by and modified by a set of OBG specialists, at two sessions.

**RESULTS:**

The specific competencies expected from students over the internship in OBG were framed within overall topics that had previously been determined and listed: healthcare, decision-making, communication and interpersonal relationships, management and organization of the Brazilian National Health System (Sistema Único de Saúde, SUS) and professionalism.

**CONCLUSIONS:**

A competency matrix that standardizes the minimum requirements that interns should be capable of putting into practice after concluding the OBG stage is a valuable tool for ensuring student performance and a fair and rigorous assessment for them, thereby seeking to train good general physicians who meet the community’s needs.

## RESUMO

**CONTEXTO E OBJETIVO:**

Este projeto de pesquisa surgiu de proposta feita aos professores pelos estudantes de um curso de medicina de uma universidade federal do Brasil, a partir de suas vivências pessoais acerca das habilidades e competências que devem ser desenvolvidas durante o estágio de Ginecologia e Obstetrícia (GO) ao longo do internato. O objetivo é desenvolver uma matriz de habilidades necessárias para a formação de um bom médico generalista no Curso de Medicina.

**TIPO DE ESTUDO E LOCAL:**

Pesquisa qualitativa do tipo exploratória realizada em uma universidade federal do Brasil.

**MÉTODOS:**

A base para o desenvolvimento dessas competências do internato em GO foi “A Matriz de Competências para o Internato Médico”, desenvolvida por Bollela e Machado. Este instrumento foi, em duas sessões, apresentado a, analisado e modificado por um grupo de especialistas em GO.

**RESULTADOS:**

As competências específicas esperadas do estudante no decorrer do internato foram enquadradas a partir de temas globais previamente determinados e listados: atenção à saúde, tomada de decisões, comunicação e relação interpessoal, gerenciamento e ordenação do SUS (Sistema Único de Saúde) e profissionalismo.

**CONCLUSÕES:**

Uma matriz de competências que padronize os requisitos mínimos que os internos sejam capazes de pôr em prática, após a conclusão do estágio em GO, é uma ferramenta valiosa para garantir o desempenho e avaliação justa e rigorosa dos estudantes, buscando a formação de um bom médico generalista, que atenda às necessidades da comunidade.

## INTRODUCTION

Medical internship is a unique process of theoretical and practical postgraduate medical training in which the aim is that physicians should become qualified for the technically differentiated practices of one of the legally recognized medical specialties.^1^ According to the Brazilian Ministry of Education, it is the “last cycle of the degree course in medicine, free of academic disciplines, during which the student must receive intensive ongoing training, under academic staff supervision, in a healthcare institution that may or may not be linked to the medical school”.^2^

The objectives of internship are presented in a general manner, without defining the basic procedures or the specific skills that students should acquire by the end of that period.^2^

Competence, from the Latin *competentia*, is the ability to do something successfully or efficiently, which means that there should be harmony between knowledge and its application. It has been used in professional training since 1973, following publication of the article “*Testing for competence rather than for ‘intelligence’*” by the American psychologist David McClelland.^3^ Currently, the concept of professional competence is defined in Brazil by the content of Article 6 of Resolution 04/99 of the National Educational Council and Basic Education Chamber (CNE/CEB), and by CNE/CEB Opinion 16/99. It is stated as the ability to mobilize, link up and put into action the values, knowledge and skills necessary for efficient and effective performance of activities required by the nature of the work.^4^ However, the nature of competence is complex: it has multiple meanings and can erroneously be confused as synonymous with skill.

Skill is often used to denote the ability to perform cognitive and/or practical acts of high complexity. The term competency is wider and includes knowledge, attitudes, cognitive skills and practices in a more holistic manner.^5^

Thus, the competencies determined for physicians cover the functions that they will be able to develop at the end of their training, thereby meeting the expectations and objectives of each stage of the medical degree. Incorporation of these skills comes from the development of technical and scientific knowledge, and from the ability to make decisions, solve problems and form attributes that, together, give the individual the skills needed to practice the profession.^6^ Thus, competency-based education focuses on physicians’ preparation for practice, guided by the needs of society and patients.^7^

In Brazil, national curriculum guidelines for courses within the field of healthcare in general, and specifically within undergraduate medical courses, have been approved. They can be considered to have resulted from significant mobilization of educators within the field of healthcare in this country, as a reflection of international trends that have led to innovations in healthcare professional training.^8^ The national curriculum guidelines have brought about changes in the curricular format of some medical courses, which were presented in grid format and were usually characterized by excessive rigidity and fragmentation, to the detriment of propositions that are more integrated and flexible as suggested in these guidelines. Thus, it can be concluded that the notion of ability, as a principle of curricular organization, would insist on attributing a “usage value” to each knowledge item. Knowledge that is delinked from practice would be treated as “without full sense”.^9^

Practical experience in this area is still relatively scarce in Brazil. However, the accumulated knowledge and experience from other countries serves as a reference for a competency-based curriculum, as outlined by Bollela in 2008, and by Bollela and Machado in 2010,^10^ for the two-year medical internship.

Obstetrics and gynecology has existed as a specialty since 1911,^11^ and it forms part of the framework of the so-called basic areas of medicine. Although treated as separate, involving different characteristics and skills among those who practice these specialties, they are linked to medical internship, even though programs are developed and enforced independently.^12^ In the beginning, obstetrics was more connected with practitioners’ experience and practice, while gynecology, being primarily surgical, followed the scientific discoveries that boosted surgery, like the advent of anesthesia and antisepsis in the 19^th^ century.^13^

In the present study, these two specialties constituted a space for reflection on the skills that should be developed during the obstetrics and gynecology stage of internship, which are necessary for training good general practitioners. The idea of this project was proposed to the course counselors by students who were part of the tutorial education program at a medical school in a federal university in Brazil. From their personal experiences, the need to conduct a study that identified the skills required today for undergraduate medical students emerged. A list of competencies for each stage of the internship of the medical course that would be necessary for training good general practitioners was drafted. The present study related to obstetrics and gynecology skills.

## OBJECTIVE

To develop a project for building the skill matrix necessary for training good general physicians in the medical course at a federal university in Brazil, detailing the skills relating to the internal medicine phase of obstetrics and gynecology (OBG).

## METHODS

This was a qualitative exploratory study using non-standardized interviews. The Competency Matrix for Medical Internship, developed by Bollela and Machado^10^ was used as the basis for building the skill set for internship in obstetrics and gynecology. This instrument was presented, in two sessions, to a set of OBG experts.

### Scenario

The scenario of this study was the medical school of a federal university in Brazil. This medical course, created in 2001, follows a new educational project and the curriculum includes integrated modules, organized according to systems and structured into 12 semesters, each comprising at least 100 days. The relevant content required for solid medical training is contained in sequential modules, longitudinal modules and internship. The medical students of this institution undertake their medical internship at Santa Casa de Misericórdia, which is the teaching hospital for this institution.

### Players

The list of competencies pertaining to the field of OBG was prepared by skilled medical professionals who were undergoing a specialization process in this specific field: residents at the teaching hospital, of whom two were former students of this medical school; representatives of teachers at this medical school; others with links to the teaching hospital; and medical academics with links to the tutorial education program at this medical school. All of these professionals participated in the development of appropriate competencies for OBG, taking into consideration that the ultimate goal was to train general practitioners.

### Theoretical reference framework

The basis for developing this skill set was the Competency Matrix for Medical Internship of Valdes Bollela. This matrix takes into consideration the different fields of knowledge and aspects of the skills expected at the end of the internship, the opportunities for learning and the evaluation methods. Although this document does not contain the particularities of each skill or field, it intentionally presents a single matrix for the two-year internship with the aim of requiring that most of the key learning objectives are identified for each general jurisdiction. Otherwise, it might not have contained the required attainments for each group of experts. From this, a set of competencies to be reached and developed within the field of OBG was developed.

### Data gathering and analysis

The process of bringing the professionals together to develop competencies through analysis of the matrix, was developed by the TEP (Tutorial Education Program) of a medical school in a federal university, in Brazil. The original matrix contained five major areas, which are listed below, together with simplified versions of their names (in parenthesis): healthcare and medical knowledge and skills (healthcare); decision-making, continuing education and learning-based practice (decision-making); communication skills and interpersonal relations (communication); leadership, management and administration and practice based on respect for the organizational order of the Brazilian National Health System (Sistema Único de Saúde, SUS) (SUS management); and Professionalism (professionalism).

The players met on two occasions. At the first meeting, the project was presented and its objectives, methods and relevance were explained. The participants then “brainstormed” suggestions for the main areas of responsibilities for the internship, and developed a thematic tree to delineate broad competencies in the field of OBG. The initial goal of these activities was to strengthen the work in the context of competencies to be exercised and avoid focusing on content, which is a common initial reaction today in discussing curriculum design today.

Once this stage was finished, the experts were divided into three groups, in which detailed analyses on the matrix were conducted in a critical and specific manner and changes were simultaneously made to the original matrix. These were recorded in notebooks by the TEP students. The domains were divided into different groups. The product was evaluated by TEP members, who brought the major changes made by the small groups together into a single list of competencies.

At the second meeting, at which the entire group of experts participated in the discussions, an overall evaluation of the unified and modified matrix was made. The matrix was drawn up, projected on a screen and distributed in copies for corrections and updates. The results relating to each of these skill areas were listed, and in this way the ones really necessary for training good general practitioners were selected and those that were very specific and only required by specialists were eliminated.

## RESULTS

This project forms part of an overall set of competencies relating to internship, and therefore there are a number of important competencies, especially with regard to professionalism, communication and organization of the healthcare system, which are common to all areas. The competencies expected from students during the internship in obstetrics and gynecology were framed from the overall issues that had previously been determined and listed as specific skills within these themes. Below, we will list some competencies from each theme. The criterion used was to present the competencies that best represented each theme within what was expected from students over the course of the internship in obstetrics and gynecology.

The overall issues listed were: healthcare, decision-making, communication, SUS management and professionalism.

Regarding healthcare, it was expected that the student should demonstrate the ability to conduct preventive care, health promotion, health protection and rehabilitation, both individually and collectively, providing attention and care as appropriate and effective for the patient (Table 1).

**Table 1.**
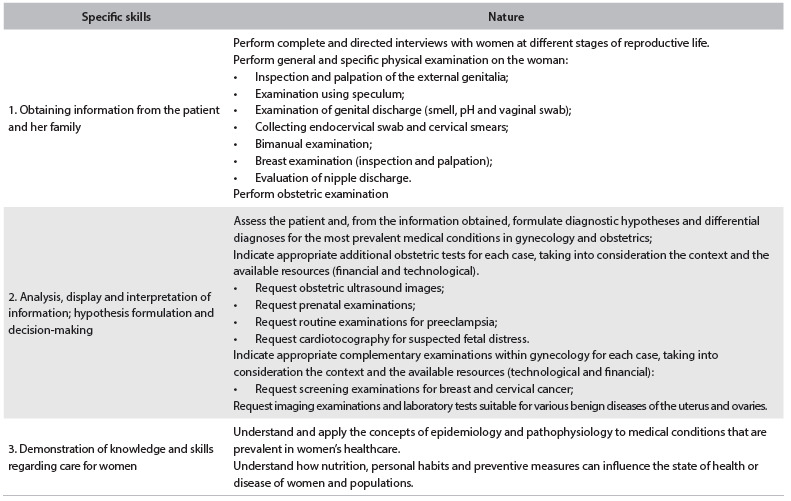
Assessment of competencies in relation to healthcare

Regarding decision-making, students were required to have the ability to make decisions regarding appropriate use, effectiveness and cost-effectiveness of the workforce, drugs, equipment, procedures and practices based on scientific evidence with application to patient care (Table 2).

**Table 2.**
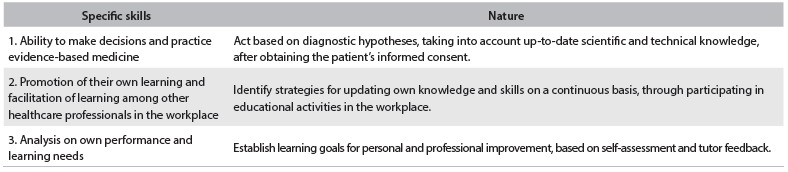
Assessment of competencies in relation to decision-making

The important competencies common to all other areas of the internship, especially the ones involving professionalism, communication and organization of the healthcare system, were framed in Table 3, in which students were expected to develop interpersonal communication skills that would result in effective information exchange and would build the doctor-patient relationship with families and other professionals. Furthermore, it was also expected that students should see themselves as members of a multidisciplinary team and be aware that, throughout their working lives, even if they were to take a leadership role in a team or healthcare service, they should maintain their commitment to the team and to the healthcare needs of the female population, with unconditional adherence to ethical principles.

**Table 3.**
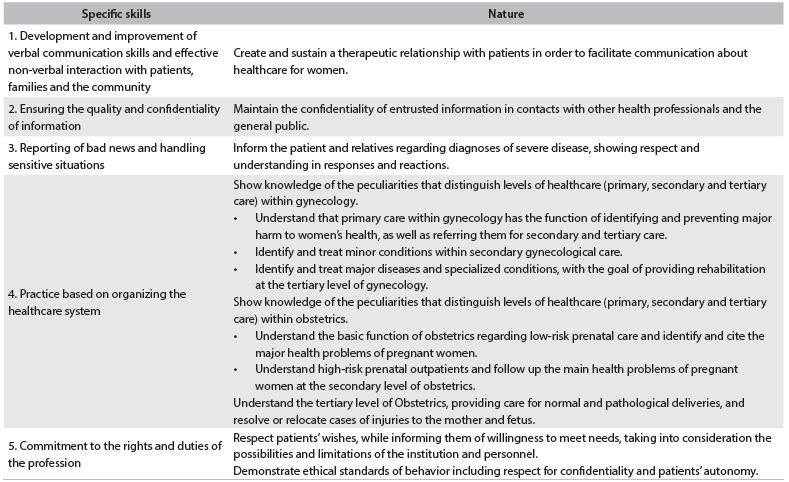
Assessment of competencies in relation to interpersonal communication, management, organizing of SUS (Sistema Único de Saúde) and professionalism

## DISCUSSION

Over the past three decades, interest in the need to define the professional competencies necessary for medical graduation and residency has greatly increased. Experiences at medical schools in the United States have demonstrated the importance of medical school participation in constructing and implementing a competency-based curriculum.^14^

For the proposed curriculum changes to actually be implemented, actions going beyond the sphere of formal documents and educational projects are essential. The governing authorities of the course need to provide strategic support for the process of change, which must necessarily involve the largest possible number of participants: teachers, students, managers of the healthcare system and academic managers.^15^

Within this interest, it is assumed that competency-based education (CBE) will lead to decreased focus on training based on time and content, to make room for an apprenticeship with greater flexibility that focuses on students.^16^ In this context, competency is understood to be the combination of personal attributes mobilized in specific contexts to achieve certain results.^17^

In this study, we report on our experience in building a “competency matrix” for OBG internship. Based on the great desire of the medical school coordinator of this federal university in Brazil to develop a manual containing the competences and abilities that newly trained general practitioners should have, TEP members, along with their tutor, took on the responsibility and challenge of developing a major project, divided into subcategories, each of which focused on an area of internship.

Because of the organization and excellence of our teaching hospital’s OBG service, already recognized here, this category was chosen for beginning the activities. The staff and residents overwhelmingly showed interest in and willingness to diligently follow the activities preestablished by the project organizers. This was fundamental to successful completion of all the steps proposed.

Initially, the activities seemed quite challenging, since they involved working directly with experts within the field and the suggested competencies were quite specific. The thematic tree was proposed precisely with the aim of broadening the experts’ views, so that they would start to think in overall terms, going from broad competency to detailed competencies.

The final matrix was a great and welcome surprise to TEP members and tutors, given that despite being the first specialty to participate in the workshops, it was the one that needed least interference from students, through removing excesses and adding what had been forgotten. Thus, the parameters defined were good for subsequent workshops, and also provided an opportunity to correct mistakes and deficiencies in the project methodology.

The OBG staff were now interested in the outcome from this matrix and evaluated the possibility of putting it into practice soon, along with the internship manual, since the matrix clarified the competencies and abilities that were expected from learners. These could be described in terms of learning objectives, and also as a method for evaluating what was actually achieved in practice, since the tests currently used are insufficient to ensure evaluation of essential competencies such as communication skills, ability to take a good clinical history and ability to conduct a physical examination, as well as teamwork.

The field of OBG is vast and multifaceted, and therefore a matrix of competencies that standardizes the minimum requirements that interns need to be able to put into practice after concluding their internship in OBG is a valuable tool for ensuring performance and fair and rigorous evaluation among students.

Studies of the same importance are being conducted in the United States, with the objective of developing operational evaluation tools regarding residents’ knowledge, in order to measure surgical cognitive competence as an independent component of general clinical competence among obstetrics and gynecology trainees.^18^ The guidelines developed by the Association of Academic Professionals in Obstetrics and Gynecology of Canada describes their expected search requirements, so that all OBG residents can successfully complete residency programs in Canada.^19^

Many leaders and educators now recognize the need to help institutions to learn, codify and understand their curriculum, thereby making it easier to put into operation. This should be done with active participation by medical school members, including interested students, residents, interns and their preceptors. This movement supports the methodology used in our study.^20^

There are few studies on interactions, during medical training, among undergraduate students, medical residents and teaching staff. Such interactions contribute towards development of a curriculum that does not present conflicts either for students or for teachers.^21^ In the literature, it is emphasized that if the medical curriculum is constructed in isolated environments with few interaction networks and without any effective integration, this leads to a curriculum of subordination, rather than exchange of knowledge, with consequent difficulties for all parties involved.^22^^,^^23^ Our study went against this subordinating methodology, given that in order to construct competencies, it relied on undergraduate students, residents and preceptors, thereby strengthening bonds through experiences.

This study has limitations that should be highlighted. If on the one hand it has great internal validity for one medical school in a federal university in Brazil, its external validity needs to be evaluated, since designing and implementing a curriculum are specific contexts that can vary greatly depending on the characteristics and conditions of each institution. The changes in evaluation proposed for the OBG service still need to be measured through satisfaction surveys among students and teachers, and also need to be approved at a meeting of the collegiate body, for it to then be put into practice.

Joint construction of the curriculum through assessment of competencies within obstetrics and gynecology is intended to provide teachers and students with an innovative teaching proposal, with coexisting impacts on teaching and evaluation of the theoretical and practical content within the field of study. It aims to provide training in competencies and to prepare physicians to gather the necessary competencies for the training of good general practitioners that meet the needs of society as a whole.

## CONCLUSIONS

In conclusion, this study has shown that it is possible, with students, preceptors and physicians of the institution, build a theoretical model of curriculum for the internship in Obstetrics and Gynecology, competency-based, containing the main guidelines of the SUS, important for the training of the general practitioner, who will soon be applied in daily practice course and expanded to other areas of medical knowledge.
